# Biological Ageing Acceleration and Functional Capacities Across the Lifespan in the INSPIRE‐T Cohort

**DOI:** 10.1002/jcsm.70046

**Published:** 2025-08-15

**Authors:** Juan Luis Sánchez‐Sánchez, Bruno Vellas, Sophie Guyonnet, Paul Bensadoun, Jean‐Marc Lemaitre, Matias Fuentealba Valenzuela, Fabien Pillard, Yves Rolland, David Furman, Philipe de Souto Barreto

**Affiliations:** ^1^ IHU HealthAge Toulouse France; ^2^ Institut du Vieillissement Centre Hospitalo‐Universitaire de Toulouse Toulouse France; ^3^ CERPOP UMR1295 University of Toulouse III, Inserm, UPS Toulouse France; ^4^ INSERM IRMB UMR1183, Hôpital Saint Eloi Université de Montpellier Montpellier France; ^5^ Buck Artificial Intelligence Platform, the Buck Institute for Research on Ageing Novato USA; ^6^ Unité de Médecine du Sport, Clinique Universitaire du Sport, Hôpital Pierre Paul RIQUET (Centre Hospitalo‐Universitaire) Toulouse France; ^7^ Centre RESTORE (Geroscience and Rejuvenation Center), UMR1301 (INSERM)/UMR5070 (CNRS) Toulouse France; ^8^ Stanford 1000 Immunomes Project Stanford University School of Medicine Stanford California USA

**Keywords:** age acceleration, ageing biology, epigenetic clocks, functional ability, healthy ageing

## Abstract

**Background:**

Biological clocks are promising tools for the evaluation of biological age deviations (i.e., positive/negative acceleration). Here, we explored the associations of biological age acceleration (BAA) assessed by Horvath's, Hannum's, PhenoAge, and GrimAge epigenetic clocks, as well as the iAge inflammation‐based clock, with functional capacities across adulthood and tested if chronological age and sex moderate these associations.

**Methods:**

Cross‐sectional analysis was conducted with baseline (2019–2021) data from 1014 participants (age range 20–104 years old, 62.82% female) drawn from the Inspire Translational Human cohort, a community‐based program in South‐West France. Physical capacity endpoints included the five‐time sit‐to‐stand test (5‐STS), the Short Physical Performance Battery (SPPB), the 30‐s chair stand test (30‐s CST), maximum oxygen uptake (VO2max) and isokinetic muscle strength (IMS). Multivariate linear regression was used to explore the associations of BAA (with and without interacting with chronological age or sex) with functional capacity endpoints.

**Results:**

A total of 1014 individuals with available data on BAA and functional capacities were included (median age 64, IQR = 49–78, 62.82% female). GrimAge was the clock that more strongly correlated with functional capacities. Higher GrimAge BAA was associated with worse 5‐STS (β = 0.25, 95% CI = 0.07, 0.43; *p* = 0.002), SPPB (β = −0.10, 95% CI = −0.18, −0.02; *p* = 0.019) and VO2max (β = −1.17, 95% CI = −1.81, −0.52; *p* < 0.001) across the whole adulthood. When the moderation effect of age was explored, BAA acceleration assessed by GrimAge was associated with worse 30‐s CST in early adulthood. Increased iAge BAA was associated with poor SPPB and 5‐STS at older age, whereas Horvath's BAA correlated with a decline in 30‐s CST.

**Conclusions:**

Among four DNA methylation epigenetic clocks and one inflammatory clock, our study shows that GrimAge is the biological ageing clock that best associates with different measures of functional capacity, from young to older adulthood.

## 1 Introduction

Ageing is associated with a decline in individual capacities, eventually leading to the onset of disability [1]. Therefore, the maintenance of functional ability, and specifically physical capacities, over the life course has become the core of healthy ageing [2, 3, 4, 5]. Age‐related declines in functional capacities are the reflection of the accumulation of molecular and cellular changes over the life course [6, S1]. Importantly, the rate of functional loss seems to vary widely between subjects of the same chronological age, suggesting that the trajectories in the age‐related decline of function might not be simply the result of the passing of time but one of the expressions of the acceleration of biological ageing [7, S2]. With the recent improvement in the mapping of the molecular pathways involved in ageing, there is a growing interest in understanding the mechanisms underlying its deviations, as well as the identification of biological age–based biomarkers able to inform about functional trajectories [8].

Among them, the estimation of biological age by means of biological clocks appears highly promising [9, S3]. These biomarkers were developed to estimate the biological age of an individual from the assessment of central mechanisms implicated in ageing, such as the methylation patterns of specific genomic locations or levels of inflammatory markers in blood [10, 11].

Changes to DNA methylation (DNAm) patterns are one of the epigenetic modifications related to ageing. DNAm consists of the covalent linkage of a methyl group to the 5′ position of the cytosine ring in a cytosine‐phospho‐guanine (CpG) dinucleotide of the DNA strand [12]. Ageing is largely associated with extended hypo‐methylation/hyper‐methylation of CpGs across the genome, and the analysis of these patterns by means of mathematical algorithms (epigenetic clocks) allows for the computation of an individual biological age estimate [13]. Biological ageing acceleration (BAA), understood as the difference in DNAm age compared to the chronological age of an individual, captured through biological clocks, has been widely linked to the onset of age‐related diseases and the risk of death [14, 15, S4, S5].

Low‐grade chronic inflammation is also considered one of the hallmarks of ageing and has been linked to several adverse health outcomes in humans, as well as to multimorbidity [16, 17, S6]. Following the principles of epigenetic clocks, Sayed et al. developed a biological clock based on the immune ageing profile of the individual, assessed by the levels of blood immune biomarkers identified by deep‐learning methods, and showed its relationship with multiple ageing phenotypes (including cardiovascular ageing, immune decline and frailty) and extraordinary survival [11].

However, the ability of biological age clocks to predict physical capacity outcomes, core components of functional ability and healthy ageing, remains unclear [18, S7, S8]. In addition, most of the scarce evidence investigating associations between BAA and functional capacities is restricted to samples of older adults [9, S9]. However, biological ageing–related mechanisms certainly start early in life [19]. In addition, both methylation and inflammatory age–related processes are dynamic, and their associations with functional changes may be different in young, middle‐age, and older adulthood. Therefore, there is a current need for a better understanding of the link between BAA (increased/decreased biological age acceleration [BAA]) and functional ageing over the whole adult life span [9].

Herein, we investigate the associations between BAA, assessed by blood DNAm patterns and inflammatory burden–based clocks and functional capacity, and study if these associations vary across the adult lifespan. We hypothesise that the increased BAA (meaning that the individuals are biologically older than their chronological age) will be associated with worse performance across adulthood, with stronger associations observed with increasing age.

## 2 Methods

### 2.1 Data Source

The present work is a cross‐sectional analysis conducted in the context of the INSPIRE research program, a geroscience platform devoted to (1) identifying markers of biological ageing in both human and animal models and (2) implementing the World Health Organization–supported Integrated Care for Older People program in the clinical scenario. A detailed description of the INSPIRE Translational Human cohort can be found elsewhere [20]. This study followed the Strengthening the Reporting of Observational Studies in Epidemiology (STROBE) guideline [S10].

### 2.2 Study Population

This study uses baseline (2019–2021) data from the Inspire Translational Human cohort, which included 1014 volunteers from the community recruited in South‐West France. Men and women aged ≥ 20 years and affiliated to the French Social Security System were included. No further eligibility criteria were applied, apart from life expectancy < 5 years (< 1 year for disabled older adults) and deprivation of liberty due to judicial and/or administrative reasons, or under current guardianship. All participants signed informed consent forms prior to inclusion. Analyses are restricted to individuals with available data on any of the exposures/endpoints investigated.

### 2.3 Biological Ageing Measures

#### 2.3.1 Epigenetic Clocks

Genomic DNA was extracted from frozen blood samples from the INSPIRE‐T cohort using the Qiagen DNeasy Blood & Tissue kit (Qiagen N.V., Vienlo, the Netherlands). After sample qualification, the Genomic DNA was bisulphite converted, and DNA methylation profiled according to the manufacturer's instructions using the EPIC Infinitum array (Illumina Inc., San Diego, USA). For each CpG locus, methylation levels (β values) ranging from 0 (completely unmethylated) to 1 (completely methylated) were calculated using Partek Genomics Suite software (Partek Inc., Chesterfield, USA).

Methylclock R package [S11] was used to calculate the following three clocks: Horvath's clock (353 CpGs) [10], Hannum's clock (71 CpGs) [21] and PhenoAge clock (513 CpGs) [14]. A fourth epigenetic clock named GrimAge clock (1030 CpGs) was calculated as described in the original paper [22].

#### 2.3.2 Inflammatory Clock

Serum samples were diluted threefold in Luminex assay buffer and run on a Luminex L200 with a custom ProCartaPlex Luminex kit (Thermo Fisher, Santa Clara, USA). For quality control, assay chex beads were added to each well (Radix Biosolutions, Georgetown, USA). Samples were run in duplicate and incubated overnight at 4°C with the Luminex beads. The trimmed mean intensity values were averaged for each sample. The trimmed distribution represents the events that were collected for an individual test in a single sample, with the lowest 5% and highest 5% of the data points removed. Analyte levels were transformed to the same scale as the Standford 1KIP data [11] using protein standards provided with the Luminex kit on each plate and those same standards run on a bridge plate containing samples that have been run on a bridge plate with samples from 1KIP. We conducted all analyses using the average trimmed mean intensity values. One linear regression model was trained on the top five analytes (CCL11, CXCL1, CXCL9, IFNG and TRAIL) contributing the most to the marker to predict iAge from the INSPIRE‐T cohort.

### 2.4 BAA Computation

BAA was calculated as the residuals of the regression of DNAm age defined by the different epigenetic clocks or iAge on chronological age, further adjusted by the cell counts (neutrophils, basophils, monocytes, lymphocytes and eosinophils) in the case of Horvath's, Hannum's and PhenoAge clocks [S12].

### 2.5 Outcome Measures

Functional capacity was measured using function‐ and performance‐based physical tests: the five‐time Sit‐to‐Stand test (5‐STS), the Short Physical Performance Battery (SPPB), the 30‐s chair stand test (30s‐CST), lower limb isokinetic muscle strength (IMS) and the cardiorespiratory fitness represented by V̇O2max (ml.kg^−^^1^.min^−1^) value attained in an incremental cardiopulmonary exercise test performed on an ergo cycle. IMS and V̇O2max were measured in a subgroup of participants (*n* = 239 [23.6%] and *n* = 245 [24.16%], respectively) of the original study in a separate session. Lower limb muscle power was estimated through the 5‐STS test, which was performed on a standard chair without armrests. Time in seconds used to perform the task was registered [S13]. Physical function was assessed by means of the SPPB. The SPPB combines the assessment of the 4‐m gait speed (GS) test, an incrementally difficult balance test and the 5‐STS test. Each domain is scored from 0 to 4, with total scores ranging between 0 and 12 [S14]. The 30s‐CST was used as a marker of lower limb muscle power/fatigability. The number of completed sit‐to‐stand cycles was registered [S15]. Lower limb isokinetic muscle strength (IMS) was used as a marker of lower limb dynamic muscle strength by using the Biodex dynamometer (Biodex System 3, Shirtley, NY, USA) at an angular velocity of 60° per second. Maximum value (in N·m) in a five‐repetition test was registered [S16, S17]. Finally, the cardiorespiratory fitness was assessed by means of the determination of maximal aerobic capacity, represented by V̇O2max (ml.kg^−1^.min^−1^) value attained in an incremental cardiopulmonary exercise test performed on an ergo cycle. The participant started cycling for 3 min with an initial workload set at 20% of the theoretical maximum aerobic power derived from Wasserman's equation. Afterwards, the workload was increased by 10% of the theoretical power reserve every minute until exhaustion. Cardiac activity and blood pressure were continuously monitored throughout the test using an electrocardiogram and a sphygmomanometer. The validated testing procedure was stopped when the following criteria of maximality were met: (1) Maximal heart rate measured at exhaustion was superior to 90% of the age‐predicted maximal heart rate, (2) respiratory exchange ratio (RER) superior to 1.1 and (3) insufficient cycling rate [S18, S19].

### 2.6 Confounders

Potential confounders consisted of age, sex, comorbidities assessed by Charlson Index and the body mass index (BMI; kg.m^−2^), that were measured using standard procedures.

### 2.7 Statistical Analysis

Descriptive statistics (mean/median and standard deviation/interquartile range or frequencies and percentages, as appropriate) were used for the characterisation of the study population. Variables were compared between the whole INSPIRE Translational Human cohort and the subsamples with available data on the endpoints by means of the Student's *T*‐Test or the Mann–Whitney *U*‐test for continuous variables, depending on their normality, whereas χ^2^ was used in the case of categorical variables.

Multiple linear regression analyses were performed to explore the association between BAA determined by the biological clocks and the outcomes. Prior to that, and after graphical visualisation of our data, non‐linear associations of age and the outcomes were hypothesised and tested by the comparison of unadjusted models: one including age as a predictor (linear model), the addition of either a polynomial or cubic terms (age^2^ and age^3^) and the mobility‐related measures as dependent variables. The likelihood ratio (*p* < 0.05) test was used to select the model that best fitted the data. Accordingly, polynomial models better represented the associations of 5‐STS, SPPB and 30s‐CST with age in our sample, whereas linear models were chosen for the IMS and the V̇O2max. Then, adjusted models were fitted including age (or age^2^) and BAA assessed by the different biological clocks (Model 1). Models were further adjusted by sex, BMI and Charlson Index (Model 2).

Given the strong associations of age with functional capacities across the lifespan, associations of BAA and the endpoints were additionally explored by the inclusion of age (or age^2^) × BAA interactive terms in fully adjusted models. When the interaction reached a *p*‐value < 0.10, the Johnson–Neyman approach was used to determine the age ranges (significance areas) at which significant associations (*p* < 0.05) between BAA and the outcomes were detected. The moderation effect of age was explored by the incorporation of an age (or age^2^) × BAA × sex triple‐interaction term into the models and, when the interaction term was significant, sex‐stratified analyses were performed. Given the potential ceiling effects of the SPPB in younger individuals (Table S3), we included sensitivity analyses restricted to individuals aged 60 years and older. All the analyses were performed with the Stata 14.0 statistical package.

## 3 Results

### 3.1 Participant Characteristics

Table 1 shows the characteristics of the study sample, composed of 1014 individuals with data on BAA assessed through any of the biological clocks. Of them, varying number of individuals had data on the endpoints: 5‐STS (*n* = 994), SPPB (*n* = 996), 30‐s CST(*n* = 744), V̇O2max (*n* = 245) and IMS (*n* = 239). Figure S1 display a detailed summary of data availability for each exposure/outcome. Subjects with available data on V̇O2max and IMS did not differ in any of the variables included compared to the rest of the sample (Table S1). Whereas median age was 61.5 ± 18.9, mean biological age was 62.1 ± 15.4, 50.8 ± 15.3, 46.2 ± 17.9, 61.2 ± 15.2 and 58.01 ± 9.18 according to Horvath's, Hannum's, PhenoAge, GrimAge and iAge clocks, respectively. Table S4 reports the error metrics of the different biological ageing clocks.

**TABLE 1:** Characteristics of the included participants.

Characteristics	Whole sample (*n* = 1014)			Women (*n* = 637)			Men (*n* = 377)		
Variable	Sample size	Statistic	Range	Sample size	Statistic	Range	Sample size	Statistic	Range
Women, No. (%)	1014	637 (62.82%)	—						
Age, y	1014	64 (49–78)	20–102	637	63 (48–76)	20–100	377	67 (51–79)	20–102
Height, m	1012	1.65 (0.10)	1.34–1.95	636	1.6 (1.56–1.65)	1.34–1.8	376	1.74 (1.69–1.79)	1.45–1.95
Weight, kg	1012	68.83 (14.12)	38–142	635	62 (55–69)	38–122	377	77.3 (69.5–86)	51–142
Body mass index, kg. m^−2^	1011	25.06 (4.29)	15.62–45.78	635	23.8 (21.4–26.8)	15.62–45.78	376	25.65 (23.42–28.15)	18.1–40.3
Charlson Index	1014	2 (1–4)	0–11	637	2 (0–4)	0–11	377	3 (1–4)	0–11
Smoking Index	776	0 (0–35)	0–2080	501	0 (0–5)	0–1960	275	0 (0–90)	0–2080
Horvath's DNAm Age, y	1002	62.14 (15.42)	18.89–101.30	629	58.4 (15.3)	18.9–69.4	373	62.9 (15.2)	21.0–101.3
Hannum's DNAm Age, y	1002	50.81 (15.30)	13.18–96.14	629	49.13 (15.05)	13.2–96.1	373	53.6 (15.3)	15.5–88.2
PhenoAge DNAm Age, y	1002	46.25 (17.87)	−4.84, 86.64	629	44.7 (17.6)	−4.84‐82.11	373	48.9 (18.0)	−0.9‐86.6
GrimAge DNAm Age, y	999	61.17 (15.23)	25.09–97.47	627	59.3 (14.8)	25.1–91.2	372	64.4 (15.4)	27.6–97.5
iAGe Age, y	1001	58.01 (9.18)	30.53–82.26	628	57.7 (9.2)	37.3–82.3	373	58.6 (9.14)	30.5–82.0
5‐STS, s	994	9.05 (2.88)	3.34–27	624	9.0 (2.9)	4–27	370	9.1 (2.9)	3.34–25
SPPB score	996	12 (12–12)	1–12	626	11.4 (12–12)	1–12	370	11.6 (12–12)	3–12
30‐s CST, n	744	15.83 (4.70)	4–40	481	15.7 (4.4)	4–31	263	16.0 (5.2)	6–40
ISM, N·m	239	103–15 (43.01)	29–244	130	81.5 (28.2)	29–177	109	129 (43.4)	52–244
V̇O2max, ml.kg^−1^.min^−1^	245	25.47 (8.50)	12–57	139	22.4 (6.0)	12–42	106	29.5 (9.6)	13–57

Abbreviations: DNAm, DNA methylation; IMS, isokinetic muscle strength; SPPB, Short Physical Performance Battery; V̇O2max, maximum oxygen uptake; 5‐STS, five‐time sit‐to‐stand test; 30‐s CST, 30‐s chair stand test.

### 3.2 Association Between BAA and Functional Ageing

In age (or age^2^), sex, BMI and Charlson Index‐adjusted models positive BAA, representing a biological age older than that expected from chronological age, were associated with worse performance in the 5‐STS, SPPB and V̇O2max when defined by GrimAge and the 30‐CST when measured by means of Horvath's DNAm age (Tables 2 and 3). No further significant associations were found. Results of sensitivity analyses of associations of BAA with SPPB restricted to older adults (≥ 60 years) are reported in Table S5.

**TABLE 2:** Associations between biological age acceleration according to the different biological clocks and 5‐STS, SPPB and the 30‐s CST.

	5‐STS			SPPB			30‐s CST		
BAA	*n*	β^a^ (95% CI)	*p*	*n*	β (95% CI)	*p*	*n*	β (95% CI)	*p*
Horvath's									
Model 1	983	0.01 (−0.16, 0.18)	0.888	984	−0.01 (−0.09, 0.06)	0.718	735	**−0.31 (−0.62, −0.002)** ^**b**^	**0.048**
Model 2	982	−0.006 (−0.17, 0.16)	0.940	904	−0.02 (−0.09, 0.06)	0.630	719	**−0.32 (−0.62, −0.02)**	**0.039**
Hannum's									
Model 1	983	0.01 (50.14, 0.18)	0.866	984	0.03 (−0.05, 0.10)	0.510	735	−0.15 (−0.45, 0.14)	0.312
Model 2	982	0.02 (−0.14, 0.18)	0.811	983	0.01 (−0.06, 0.09)	0.700	719	−0.21 (−0.50, 0.07)	0.148
PhenoAge									
Model 1	983	0.05 (−0.11, 0.22)	0.536	983	−0.01 (−0.09, 0.06)	0.740	735	−0.26 (−0.56, 0.03)	0.082
Model 2	982	0.01 (−0.15, 0.17)	0.929	983	0.01 (−0.07, 0.08)	0.860	719	−0.24 (−0.53, 0.05)	0.103
GrimAge									
Model 1	980	**0.29 (0.13 0.46)**	***p* < 0.001**	981	**−0.10 (−0.18, −0.03)**	**0.009**	732	−0.27 (−0.59, 0.05)	0.096
Model 2	979	**0.25 (0.07, 0.43)**	**0.005**	980	**−0.10 (−0.18, −0.02)**	**0.019**	716	−0.29 (−0.63, 0.05)	0.095
iAge									
Model 1	982	**0.07 (0.0003, 0.001)**	**0.002**	983	−0.04 (−0.12, 0.05)	0.415	739	−0.13 (−0.49, 0.21)	0.439
Model 2	981	0.16 (−0.03, 0.34)	0.096	982	−0.08 (−0.16, 0.01)	0.082	723	−0.26 (−0.60, 0.07)	0.130

Abbreviations: DNAm, DNA methylation; SPPB, Short Physical Performance Battery; 5‐STS, five‐time sit‐to‐stand test; 30‐s CST, 30‐s chair stand test.

^a^ β‐coefficient corresponds to the increase in 1‐SD in the BAA.

^b^ Significant associations are displayed in bold.

**TABLE 3:** Associations between biological age acceleration according to the different biological clocks and IMS and V̇O2max.

	IMS			V̇O2max		
BAA	*n*	β^a^ (95% CI)	*p*	*n*	β (95% CI)	*p*
Horvath's						
Model 1	237	−2.45 (−3.17, 8.07)	0.392	243	−0.11 (−1.11, 0.89)	0.832
Model 2	237	−2.53 (−7.02, 1.95)	0.267	243	−0.57 (−1.32, 0.18)	0.136
Hannum's						
Model 1	237	−1.81 (−3.65, 7.28)	0.514	243	**0.99 (0.03, 1.94)**	**0.042**
Model 2	237	−2.61 (−6.97, 1.76)	0.241	243	0.26 (−0.48, 0.99)	0.489
PhenoAge						
Model 1	237	−1.69 (−3.71 7.09)	0.458	243	−0.46 (−1.41, −0.48)^b^	0.336
Model 2	237	−2.55 (−6.84, 1.74)	0.244	243	−0.55 (−1.27, 0.16)	0.130
GrimAge						
Model 1	237	8.17 (3.91, 12.42)	0.949	243	0.18 (−0.61, −0.96)	0.655
Model 2	237	−0.49 (−4.24, 3.26)	0.797	237	**−1.17 (−1.81. ‐0.52)**	***p* < 0.001**
iAge						
Model 1	232	−3.89 (−9.42, 1.63)	0.151	237	0.33 (−0.54, 1.20)	0.457
Model 2	232	−4.18 (−8.54, 0.19)	0.061	237	0.19 (−0.47, 0.86)	0.563

Abbreviations: IMS, isokinetic muscle strength; V̇O2max, maximum oxygen uptake.

^a^ β‐coefficient corresponds to the increase in 1‐SD in the BAA.

^b^ Significant associations are displayed in bold.

**FIGURE 1:**
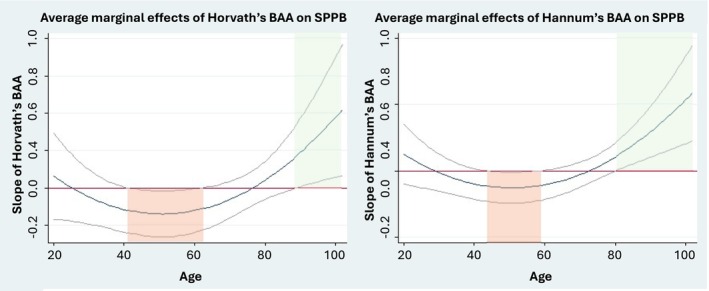
Moderation of age on the association between BAA by Horvath's and Hannum's clocks and the SPPB. Average marginal effects correspond to the value of the β‐coefficient at different values of age given the data. Red and green areas correspond to the associations between 1‐SD increase in the BAA and worse and better performance in the outcome, respectively.

**FIGURE 2:**
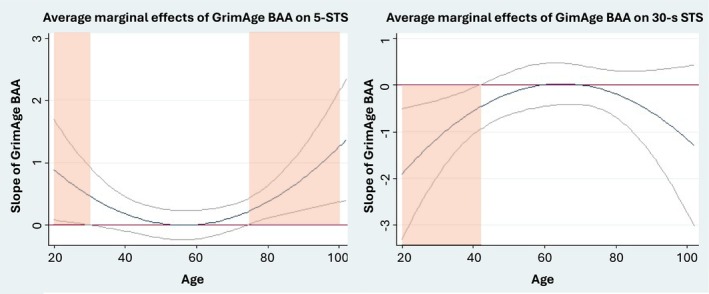
Moderation of age on the association between BAA by GrimAge clock and the 5‐STS and the 30‐s CST. Average marginal effects correspond to the value of the β‐coefficient at different values of age given the data. Red and green areas correspond to the associations between 1‐SD increase in the BAA and worse and better performance in the outcome, respectively.

**FIGURE 3:**
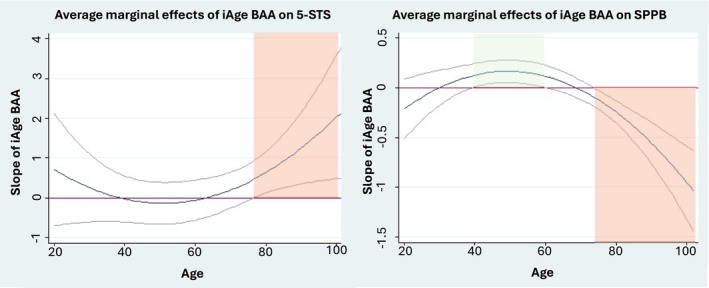
Moderation of age on the association between BAA by iAge clock and the SPPB and the 5‐STS. Average marginal effects correspond to the value of the β‐coefficient at different values of age given the data. Red and green areas correspond to the associations between 1‐SD increase in the BAA and worse and better performance in the outcome, respectively.

**FIGURE 4:**
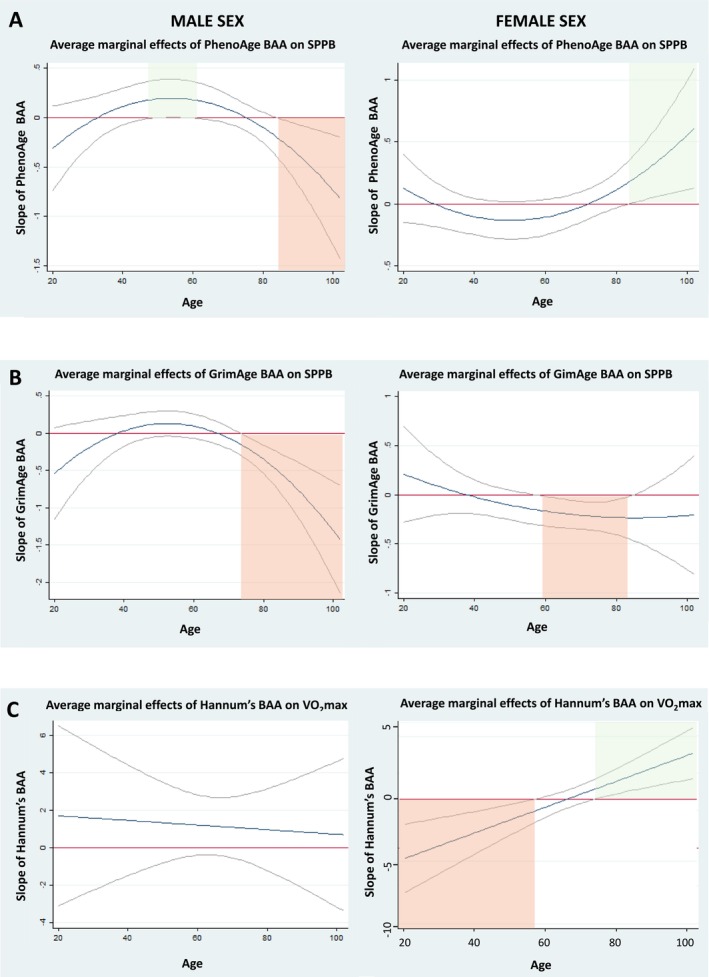
Moderation of age on the association between BAA and functional outcomes by sex. (A–C) Average marginal effects correspond to the value of the β‐coefficient at different values of age given the data. Red and green areas correspond to the associations between 1‐SD increase in the BAA and worse and better performance in the outcome, respectively.

We found significant *age* (*or age* ^*2*^) moderation effects at the *α* = 0.1 level, indicating modification of the associations by age, on the associations between BAA and 5‐STS when BAA was defined by GrimAge DNAm and iAge clocks; on the association with the SPPB score when assessed by Horvath's and Hannum's DNAm age and iAge; and on the association with 30‐CST when assessed by GrimAge (Table S2). The Johnson–Neyman approach showed significant associations between increased BAA defined by Horvath's and Hannum's and worse performance in SPPB at middle age (age range = 43–63 and 45–59, respectively) and better SPPB performance at very old age (age range = 91–102 and 82–102, respectively, Figure 1). GrimAge DNAm increased BAA was associated with worse performance in both the 5‐STS at two age ranges (20–29 and 76–102 years of age) and the 30‐STS at the age range of 20–44 years (Figure 2). Finally, increased BAA assessed by iAge showed a significant association with better performance in the SPPB at middle age (45–66 years of age) and worse performance in the 5‐STS and the SPPB at old age (with areas of significance covering the 77–102 and 67–102 age ranges, respectively) (Figure 3). Analyses exploring the moderation effect of sex on the associations showed a significant triple *age* (*or age* ^*2*^) *× BAA × sex* interaction regarding the association between PhenoAge BAA and the SPPB, GrimAge and SPPB, and Hannum's and VO_2_max. Specifically, PhenoAge BAA was associated with higher SPPB score in men in the age range of 51–58 and in women between 84 and 102 years of age and worse SPPB score in men older than 84 (Figure 4A). GrimAge BAA was associated with worse performance in the SPPB in men older than 74 years and in women in the age range of 58–86 (Figure 4B). Finally, BAA assessed by Hannum's was linked to worse VO_2_max in women aged 20–57 and better VO_2_max in those aged 75–102 (Figure 4C).

## 4 Discussion

Our cross‐sectional study is among the first to explore the associations between BAA, assessed by means of epigenetic and inflammatory clocks, and physical capacities across adulthood (20–102 years of age). Overall, our results suggest an association between BAA, defined by the GrimAge DNAm–based clock, and worse performance on functional capacity tests. In addition, BAA assessed by the iAge clock, representing the inflammatory burden of the individual, was associated with worse performance in the SPPB and 5‐STS at old age. Interestingly, our results suggest that the direction of the association between BAA and physical capacities might change across adulthood, with periods in which higher BAA is associated with better outcomes. This mirrors traits that follow an antagonistic pleiotropic scheme where biological features that can be beneficial early in the life of an organism are harmful later in life [23].

With the current shift in the care of older adults' health, in which maintenance of individual physical and mental capacities is prioritised, the understanding of the mechanisms driving healthy/accelerated biological ageing is a current priority [24]. Our study adds to emerging evidence on the association of promising biological age estimates and physical capacities and, to the best of our knowledge, investigates for the first time the influence of BAA on this relevant outcome across the whole adulthood, from young to very old ages. The results of this study mirror those of previous research on the link of DNAm‐based clocks and individual capacities, pointing to an outperformance of GrimAge in predicting physical functioning compared to its epigenetic first‐ and second‐generation clock pairs [15, 25]. For example, Föhr et al., using data from the female sample of the Finnish Twin Study on Ageing (*n* = 413, mean age 68.6 ± 3.4), and on the same four epigenetic clocks in our study, showed a unique association of increased GrimAge BAA with a poor performance in the Up‐and‐Go Test and the 6‐min walking test cross‐sectionally and greater decline in the performance of the Up‐and‐Go Test, 10‐m gait speed test, knee extension strength and the 6‐min walking test over the 3‐year follow‐up; no associations were found for the other biological age biomarkers [25]. Our study, including both men and women and encompassing human adulthood from age 20 to 100, showed that BAA assessed by GrimAge was associated with worse performance in the SPPB, 5‐STS and lower cardiorespiratory fitness. An increased BAA assessed by Horvath's DNAm was only associated with worse performance in the 30‐s CST, whereas PhenoAge DNAm BAA was associated with a lower cardiorespiratory fitness.

The superior ability of GrimAge to predict the physical capacities of the individual has been widely attributed in the literature to the fact that this epigenetic clock was trained on healthspan measures and might be more suitable to capture phenotypes of ageing compared to its pairs, originally developed to predict chronological age or mortality [10, 14, 26]. In fact, it has been argued that first generation clocks (i.e., Horvath's and Hannum's DNAm clocks), by focusing on calendar age, may in fact exclude patterns of CpGs methylation relevant to biological age and, therefore, might be imperfect surrogates of an individual's ageing rate [13, 14] compared to its pairs, originally developed to predict chronological age or mortality [10, 13]. In agreement with this, a recent pre‐print showed that a novel blood‐based epigenetic clock trained on the novel construct of intrinsic capacity measures outperforms other epigenetic clocks in predicting both all‐cause and cardiovascular‐related mortality, suggesting that clocks trained against relevant health parameters might be more informative than chronological age‐trained ones [27].

Despite some studies linking the increase in first‐generation clocks DNAm BAA with worse handgrip strength [28, 29, 30], the great bulk of the evidence points to the absence of significant associations with the latter and other physical capacity–related endpoints [25, 30, 31]. In our study, we showed a weak association between BAA defined by Horvath's and Hannum's DNAm and worse SPPB scores among middle‐aged individuals. This might indicate an overlap between primary ageing (mediated by the passing of time) and acceleration of biological ageing in this age group, given that individuals displaying older DNAm‐based estimation of chronological age also show deteriorated functional abilities. Surprisingly, we also found positive associations between BAA and SPPB scores in the oldest old (> 91 and > 81 for Horvath's and Hannum's, respectively), which might be, at least partly, explained by a potential survival bias and the small sample size in this age range: Given that these first‐generation DNAm clocks reflect chronological age, it is possible that subjects who have reached very old age and accept to participate in a research study might be those with exceptional good health according to their age and, therefore, presenting with outstanding high levels of physical performance.

In addition, whereas PhenoAge DNAm clock, developed against a composite measure of age and nine ageing‐related clinical markers [14], has been reported to be associated with early mortality and age‐related diseases such as cancer, its ability to capture functional ageing of an individual might be limited according to our results and those of previous research [15, 25, 31, 32]. Only the study of Maddock and collaborators showed a marginal link between BAA assessed by PhenoAge DNAm and worse evolution in the handgrip strength test [31], not observed in the rest of the studies.

To our knowledge, our study is the first one exploring the link between an inflammation‐based clock (iAge) and physical capacities in a sample different from the one in which this marker was developed and validated [11]. Our results point to an association of BAA captured by iAge and worse SPPB and 5‐STS at old age, which reinforces the role of inflammation as a master actor of acceleration in functional ageing [33]. The restriction of the link to older age might result from the late‐life onset of immunosenescence, the increased importance of cell senescence, the pro‐inflammatory senescence–associated secretory phenotype and the collapse of anti‐inflammatory mechanisms linked to ageing [34, 35]. Taken together, this leads to the presence of low‐grade chronic inflammation, a fundamental driver of the loss of both muscle mass and function and, hence, physical capacity, with ageing [34, 35]. In addition, we found a slight association between higher BAA by iAge and better SPPB scores in middle‐aged individuals. This unexpected finding might be due to the ceiling effects of the SPPB in this age range, with only two (1.03%) of the 194 individuals in this age range presenting with an SPPB score lower than 12, having a lower iAge (55.68 ± 11.74 vs. 58.62 ± 8.75) than individuals with an SPPB of 12. On the other hand, it has been proposed that inflammation follows rules of antagonistic pleiotropy with different effects across the lifespan, a positive stress‐response effect in younger individuals and a negative impact in the context of age‐related low‐grade chronic diseases [34, 36].

We showed moderation effects of sex on the associations between BAA and certain outcomes. Previous observational research suggests an accelerated biological ageing rate among men compared to women [37], which might explain our observations. However, no study has explored its impact on functional abilities and physical capacities, in particular. Therefore, further research should explore the role of sex on the link between BAA and the physical capacities.

Our study should be interpreted in light of some limitations. The cross‐sectional design limits our ability to completely rule out inverse causality. Despite the logical understanding of physical capacities as expressions of underlying physiological processes such as biological ageing, it is also plausible that better physical performance might allow engagement in healthier lifestyles (i.e., greater physical activity) and therefore lower biological age. Longitudinal studies might shed light on the causal direction of the observed association. Furthermore, in our study, we estimated the BAA based on blood samples; differential epigenetic age across tissues might have distorted our results [38, 39]. Also, the wide range of age in our study might have limited sample sizes for specific age groups. In addition, the sample of our study might not be representative of the general oldest old population, and inferences should be performed carefully. For SPPB, a ceiling effect might have altered the association among young adults. Also, in the case of iAge‐determined BAA, the reliance on cross‐sectional assessment of blood cytokines might have led to overestimation in cases of transient stress such as recent participation in strenuous physical exercise or infection. Finally, given the observational nature of our work, residual confounding cannot be ruled out. On the other hand, despite the relatively small sample size in the 90+ age group (*N* = 22), our work presents strengths such as the relatively large sample size for investigations using biological age clocks, the inclusion of both men and women from 20 to over 100 years of age and the exploration of the interaction between age and BAA, given the tight link between the latter and the physical capacity endpoints.

The incorporation of reliable biological ageing markers capable of predicting healthspan and functional trajectories of ageing, rather than lifespan or mortality, might assist in the establishment of therapeutic targets and individual's ageing rate monitoring and serve as outcomes to test intervention effects on ageing [40]. Our study suggests the GrimAge and iAge estimators might be useful as markers of biological ageing, over chronological age– or mortality‐based counterparts. Further longitudinal and experimental research, benefiting from the refinement of biological ageing–based markers and the inclusion of physical capacity–related tests that can capture changes along the whole adulthood (from young, middle‐aged to older adults), may expand our knowledge around the ability of the former to capture individual risk of functional decline, evaluate whether changes in these biomarkers mirror the functional trajectories along the ageing process and assess the effect of interventions oriented to promote healthy ageing by interfering with the accelerated biological ageing process.

## Ethics Statement

The INSPIRE Translational Human cohort (INSPIRE‐T) was designed according to the 1964 Declaration of Helsinki and registered on clinicaltrials.gov (ID NTC04224038). Both the French Ethics Committee (Rennes and CPP Ouest V) and the French National Commission for Data Protection (Ref. No. MMS/OSS/NDT171027) approved the study protocol. This work fulfils the ethical guidelines for publishing in the *Journal of Cachexia, Sarcopenia and Muscle* [41].

## Conflicts of Interest

The authors declare no conflicts of interest.

## Supporting information


**Table S1:** Comparison of the characteristics of included individuals based on the availability of data on VO2max/IMS.
**Table S2:** Independent and interactive association of BAA and age (or age^2^) and physical capacities in the INSPIRE sample. Bold *p‐*values indicate statistical significance. IV, independent variable.
**Table S3:** SPPB scores by age group.
**Table S4:** Mean absolute error, median absolute error and root mean square error of the biological age estimated by biological clocks and chronological age in our sample.
**Table S5:** Associations between biological age acceleration according to different biological clocks and SPPB in participants ≥ 60 years.

## References

[1] P. de Souto Barreto , Y. Rolland , L. Ferrucci , et al., “Looking at Frailty and Intrinsic Capacity Through a Geroscience Lens: The ICFSR and Geroscience Task Force,” Nature Aging 3 (2023): 1474–1479. 37985720 10.1038/s43587-023-00531-w PMC12159420

[2] M. Cesari , Y. Sumi , Z. A. Han , et al., “Implementing Care for Healthy Ageing,” BMJ Global Health 7 (2022): e007778. 10.1136/bmjgh-2021-007778 PMC8860009 35185014

[3] M. Billot , R. Calvani , A. Urtamo , et al., “Preserving Mobility in Older Adults With Physical Frailty and Sarcopenia: Opportunities, Challenges, and Recommendations for Physical Activity Interventions,” Clinical Interventions in Aging 15 (2020): 1675–1690. 32982201 10.2147/CIA.S253535 PMC7508031

[4] J. L. Sánchez‐Sánchez , Y. Rolland , M. Cesari , and P. de Souto Barreto , “Associations Between Intrinsic Capacity and Adverse Events Among Nursing Home Residents: The INCUR Study,” Journal of the American Medical Directors Association 23 (2022): 872–876.e4. 34571043 10.1016/j.jamda.2021.08.035

[5] J. L. Sánchez‐Sánchez , W.‐H. Lu , D. Gallardo‐Gómez , et al., “Association of Intrinsic Capacity With Functional Decline and Mortality in Older Adults: A Systematic Review and Meta‐Analysis of Longitudinal Studies,” Lancet Healthy Longevity 5 (2024): e480–e492. 38945130 10.1016/S2666-7568(24)00092-8

[6] C. López‐Otín , M. A. Blasco , L. Partridge , et al., “The Hallmarks of Ageing,” Cell 153 (2013): 1194–1217. 23746838 10.1016/j.cell.2013.05.039 PMC3836174

[7] D. J. Lowsky , S. J. Olshansky , J. Bhattacharya , et al., “Heterogeneity in Healthy Ageing,” Journals of Gerontology. Series A, Biological Sciences and Medical Sciences 69 (2014): 640–649. 24249734 10.1093/gerona/glt162 PMC4022100

[8] S. D. Anton , Y. Cruz‐Almeida , A. Singh , et al., “Innovations in Geroscience to Enhance Mobility in Older Adults,” Experimental Gerontology 142 (2020): 111123. 33191210 10.1016/j.exger.2020.111123 PMC7581361

[9] A. Gaylord , A. Cohen , and A. Kupsco , “Biomarkers of Ageing Through the Life Course: A Recent Literature Update,” Current Opinion in Epidemiology and Public Health 2 (2023): 7–17. 38130910 10.1097/pxh.0000000000000018 PMC10732539

[10] S. Horvath , “DNA Methylation Age of Human Tissues and Cell Types,” Genome Biology 14 (2013): R115. 24138928 10.1186/gb-2013-14-10-r115 PMC4015143

[11] N. Sayed , Y. Huang , K. Nguyen , et al., “An Inflammatory Ageing Clock (iAge) Based on Deep Learning Tracks Multimorbidity, Immunosenescence, Frailty and Cardiovascular Ageing,” Nature Aging 1 (2021): 598–615. 34888528 10.1038/s43587-021-00082-y PMC8654267

[12] K. Day , L. L. Waite , A. Thalacker‐Mercer , et al., “Differential DNA Methylation With Age Displays Both Common and Dynamic Features Across Human Tissues That Are Influenced by CpG Landscape,” Genome Biology 14 (2013): R102. 24034465 10.1186/gb-2013-14-9-r102 PMC4053985

[13] R. Duan , Q. Fu , Y. Sun , et al., “Epigenetic Clock: A Promising Biomarker and Practical Tool in Ageing,” Ageing Research Reviews 81 (2022): 101743. 36206857 10.1016/j.arr.2022.101743

[14] M. E. Levine , A. T. Lu , A. Quach , et al., “An Epigenetic Biomarker of Ageing for Lifespan and Healthspan,” Ageing (Albany NY) 10 (2018): 573–591. 10.18632/aging.101414 PMC5940111 29676998

[15] C. McCrory , G. Fiorito , B. Hernandez , et al., “GrimAge Outperforms Other Epigenetic Clocks in the Prediction of Age‐Related Clinical Phenotypes and All‐Cause Mortality,” Journals of Gerontology – Series A Biological Sciences and Medical Sciences 76 (2021): 741–749. 33211845 10.1093/gerona/glaa286 PMC8087266

[16] F. Sanada , Y. Taniyama , J. Muratsu , et al., “Source of Chronic Inflammation in Ageing,” Frontiers in Cardiovascular Medicine 5, no. 12 (2018). 10.3389/fcvm.2018.00012 PMC5850851 29564335

[17] D. Furman , J. Campisi , E. Verdin , et al., “Chronic Inflammation in the Etiology of Disease Across the Life Span,” Nature Medicine 25 (2019): 1822–1832. 10.1038/s41591-019-0675-0 PMC7147972 31806905

[18] L. Ferrucci , M. Gonzalez‐Freire , E. Fabbri , et al., “Measuring Biological Ageing in Humans: A Quest,” Ageing Cell 19 (2020): e13080. 10.1111/acel.13080 PMC6996955 31833194

[19] A. A. Cohen , B. K. Kennedy , U. Anglas , et al., “Lack of Consensus on an Ageing Biology Paradigm? A Global Survey Reveals an Agreement to Disagree, and the Need for an Interdisciplinary Framework,” Mechanisms of Ageing and Development 191 (2020): 111316. 32693105 10.1016/j.mad.2020.111316 PMC7603428

[20] S. Guyonnet , Y. Rolland , C. Takeda , et al., “The INSPIRE Bio‐Resource Research Platform for Healthy Ageing and Geroscience: Focus on the Human Translational Research Cohort (The INSPIRE‐T Cohort),” Journal of Frailty and Aging 10 (2021): 110–120. 33575699 10.14283/jfa.2020.38 PMC7352084

[21] G. Hannum , J. Guinney , L. Zhao , et al., “Genome‐Wide Methylation Profiles Reveal Quantitative Views of Human Ageing Rates,” Molecular Cell 49 (2013): 359–367. 23177740 10.1016/j.molcel.2012.10.016 PMC3780611

[22] A. T. Lu , A. Quach , J. G. Wilson , et al., “DNA Methylation GrimAge Strongly Predicts Lifespan and Healthspan,” Aging 11 (2019): 303–327. 30669119 10.18632/aging.101684 PMC6366976

[23] S. N. Austad and J. M. Hoffman , “Is Antagonistic Pleiotropy Ubiquitous in Ageing Biology?,” Evolution, Medicine, and Public Health 2018 (2018): 287–294. 30524730 10.1093/emph/eoy033 PMC6276058

[24] D. R. Seals and S. Melov , “Translational Geroscience: Emphasizing Function to Achieve Optimal Longevity,” Aging 6 (2014): 718–730. 25324468 10.18632/aging.100694 PMC4221919

[25] T. Föhr , T. Törmäkangas , H. Lankila , et al., “The Association Between Epigenetic Clocks and Physical Functioning in Older Women: A 3‐Year Follow‐Up,” Journals of Gerontology. Series A, Biological Sciences and Medical Sciences 77 (2022): 1569–1576. 34543398 10.1093/gerona/glab270 PMC9373966

[26] B. H. Chen , R. E. Marioni , E. Colicino , et al., “DNA Methylation‐Based Measures of Biological Age: Meta‐Analysis Predicting Time to Death,” Aging 8 (2016): 1844–1865. 27690265 10.18632/aging.101020 PMC5076441

[27] M. Fuentealba , L. Rouch , S. Guyonnet , et al., “A Novel Blood‐Based Epigenetic Clock for Intrinsic Capacity Predicts Mortality and is Associated With Clinical, Immunological and Lifestyle Factors,” bioRxiv (2024): 2024.08.09.607252. 10.1038/s43587-025-00883-5 PMC12270914 40467932

[28] R. E. Marioni , S. Shah , A. F. McRae , et al., “The Epigenetic Clock Is Correlated With Physical and Cognitive Fitness in the Lothian Birth Cohort 1936,” International Journal of Epidemiology 44 (2015): 1388–1396. 25617346 10.1093/ije/dyu277 PMC4588858

[29] A. J. Simpkin , R. Cooper , L. D. Howe , et al., “Are Objective Measures of Physical Capability Related to Accelerated Epigenetic Age? Findings From a British Birth Cohort,” BMJ Open 7 (2017): e016708. 10.1136/bmjopen-2017-016708 PMC5695310 29092899

[30] E. Sillanpää , A. Heikkinen , A. Kankaanpää , et al., “Blood and Skeletal Muscle Ageing Determined by Epigenetic Clocks and Their Associations With Physical Activity and Functioning,” Clinical Epigenetics 13 (2021): 110. 34001218 10.1186/s13148-021-01094-6 PMC8127311

[31] J. Maddock , J. Castillo‐Fernandez , A. Wong , et al., “DNA Methylation Age and Physical and Cognitive Ageing,” Journals of Gerontology – Series A Biological Sciences and Medical Sciences 75 (2020): 504–511. 31630156 10.1093/gerona/glz246 PMC8414926

[32] S. Kabacik , D. Lowe , L. Fransen , et al., “The Relationship Between Epigenetic Age and the Hallmarks of Ageing in Human Cells,” Nature Aging 2 (2022): 484–493. 37034474 10.1038/s43587-022-00220-0 PMC10077971

[33] C. Franceschi , M. Bonafè , S. Valensin , et al., “Inflamm‐Ageing. An Evolutionary Perspective on Immunosenescence,” Annals of the New York Academy of Sciences 908 (2000): 244–254. 10911963 10.1111/j.1749-6632.2000.tb06651.x

[34] T. Fulop , A. Larbi , G. Pawelec , et al., “Immunology of Ageing: The Birth of Inflammageing,” Clinical Reviews in Allergy and Immunology 64 (2023): 109–122. 34536213 10.1007/s12016-021-08899-6 PMC8449217

[35] E. Marzetti , A. Picca , F. Marini , et al., “Inflammatory Signatures in Older Persons With Physical Frailty and Sarcopenia: The Frailty ‘Cytokinome’ at Its Core,” Experimental Gerontology 122 (2019): 129–138. 31054959 10.1016/j.exger.2019.04.019

[36] M. Goto , “Inflammageing (Inflammation + Ageing): A Driving Force for Human Ageing Based on an Evolutionarily Antagonistic Pleiotropy Theory?,” Bioscience Trends 2 (2008): 218–230. 20103932

[37] I. Yusipov , A. Kalyakulina , A. Trukhanov , C. Franceschi , and M. Ivanchenko , “Map of Epigenetic Age Acceleration: A Worldwide Analysis,” Ageing Research Reviews 100 (2024): 102418. 39002646 10.1016/j.arr.2024.102418

[38] E. E. Guevara , W. D. Hopkins , P. R. Hof , J. J. Ely , B. J. Bradley , and C. C. Sherwood , “Epigenetic Ageing of the Prefrontal Cortex and Cerebellum in Humans and Chimpanzees,” Epigenetics 17 (2022): 1774–1785. 35603816 10.1080/15592294.2022.2080993 PMC9621016

[39] A. T. Lu , Z. Fei , A. Haghani , et al., “Universal DNA Methylation Age Across Mammalian Tissues,” Nature Aging 3 (2023): 1144–1166. 37563227 10.1038/s43587-023-00462-6 PMC10501909

[40] M. Kaeberlein , P. S. Rabinovitch , and G. M. Martin , “Healthy Ageing: The Ultimate Preventative Medicine,” Science 350 (2015): 1191–1193. 26785476 10.1126/science.aad3267 PMC4793924

[41] S. von Haehling , A. J. S. Coats , and S. D. Anker , “Ethical Guidelines for Publishing in the Journal of Cachexia, Sarcopenia and Muscle: Update 2021,” Journal of Cachexia, Sarcopenia and Muscle 12, no. 6 (2021): 2259–2261. 34904399 10.1002/jcsm.12899 PMC8718061

