# Supplementary material for: Biological Ageing Acceleration and Functional Capacities Across the Lifespan in the INSPIRE‐T Cohort
Source: J Cachexia Sarcopenia Muscle. 2025 Aug 15;16(4):e70046. doi: 10.1002/jcsm.70046 (PMC12355191; doi:10.1002/jcsm.70046)
Supplement: Supplementary file 1 — Table S1: Comparison of the characteristics of included individuals based on the availability of data on VO2max/IMS. Table S2: Independent and interactive association of BAA and age (or age2) and physical capacities in the INSPIRE sample. Bold p‐values indicate statistical significance. IV, independent variable. Table S3: SPPB scores by age group. Table S4: Mean absolute error, median absolute error and root mean square error of the biological age estimated by biological clocks and chronological age in our sample. Table S5: Associations between biological age acceleration according to different biological clocks and SPPB in participants ≥ 60 years. [file JCSM-16-e70046-s001.docx]

**Supplementary references**

S1. B. K. Kennedy, S. L. Berger, A. Brunet, J. Campisi, A. M. Cuervo, E. S. Epel, C. Franceschi, G. J. Lithgow, R. I. Morimoto, J. E. Pessin, T. A. Rando, A. Richardson, E. E. Schadt, T. Wyss-Coray, F. Sierra, Geroscience: linking aging to chronic disease. *Cell* **159**, 709–713 (2014).

S2. M. Moqri, C. Herzog, J. R. Poganik, Biomarkers of Aging Consortium, J. Justice, D. W. Belsky, A. Higgins-Chen, A. Moskalev, G. Fuellen, A. A. Cohen, I. Bautmans, M. Widschwendter, J. Ding, A. Fleming, J. Mannick, J.-D. J. Han, A. Zhavoronkov, N. Barzilai, M. Kaeberlein, S. Cummings, B. K. Kennedy, L. Ferrucci, S. Horvath, E. Verdin, A. B. Maier, M. P. Snyder, V. Sebastiano, V. N. Gladyshev, Biomarkers of aging for the identification and evaluation of longevity interventions. *Cell* **186**, 3758–3775 (2023).

S3. S. Kabacik, D. Lowe, L. Fransen, M. Leonard, S.-L. Ang, C. Whiteman, S. Corsi, H. Cohen, S. Felton, R. Bali, S. Horvath, K. Raj, The relationship between epigenetic age and the hallmarks of aging in human cells. *Nat Aging* **2**, 484–493 (2022).

S4. J. D. Roberts, E. Vittinghoff, A. T. Lu, A. Alonso, B. Wang, C. M. Sitlani, P. Mohammadi-Shemirani, M. Fornage, J. Kornej, J. A. Brody, D. E. Arking, H. Lin, S. R. Heckbert, I. Prokic, M. Ghanbari, A. C. Skanes, T. M. Bartz, M. V. Perez, K. D. Taylor, S. A. Lubitz, P. T. Ellinor, K. L. Lunetta, J. S. Pankow, G. Paré, N. Sotoodehnia, E. J. Benjamin, S. Horvath, G. M. Marcus, Epigenetic Age and the Risk of Incident Atrial Fibrillation. *Circulation* **144**, 1899–1911 (2021).

S5. S. E. Johnstone, V. N. Gladyshev, M. J. Aryee, B. E. Bernstein, Epigenetic clocks, aging, and cancer. *Science* **378**, 1276–1277 (2022).

S6. A. Bektas, S. H. Schurman, R. Sen, L. Ferrucci, Aging, inflammation and the environment. *Exp Gerontol* **105**, 10–18 (2018).

S7. J. Jylhävä, N. L. Pedersen, S. Hägg, Biological Age Predictors. *EBioMedicine* **21**, 29–36 (2017).

S8. G. T. Baker, R. L. Sprott, Biomarkers of aging. *Exp Gerontol* **23**, 223–239 (1988).

S9. V. M. Vetter, C. H. Kalies, Y. Sommerer, D. Spira, J. Drewelies, V. Regitz-Zagrosek, L. Bertram, D. Gerstorf, I. Demuth, Relationship Between 5 Epigenetic Clocks, Telomere Length, and Functional Capacity Assessed in Older Adults: Cross-Sectional and Longitudinal Analyses. *J Gerontol A Biol Sci Med Sci* **77**, 1724–1733 (2022).

S10. STROBE Statement – checklist of items that should be included in reports of observational studies1 (© STROBE Initiative). *Int J Public Health* **53**, 3–4 (2008).

S11. D. Pelegí-Sisó, P. de Prado, J. Ronkainen, M. Bustamante, J. R. González, methylclock: a Bioconductor package to estimate DNA methylation age. *Bioinformatics* **37**, 1759–1760 (2021).

S12. G. Hannum, J. Guinney, L. Zhao, L. Zhang, G. Hughes, S. Sadda, B. Klotzle, M. Bibikova, J.-B. Fan, Y. Gao, R. Deconde, M. Chen, I. Rajapakse, S. Friend, T. Ideker, K. Zhang, Genome-wide Methylation Profiles Reveal Quantitative Views of Human Aging Rates. *Molecular Cell* **49**, 359–367 (2013).

S13. Coelho-Júnior HJ, da Silva Aguiar S, de Oliveira Gonçalves I, et al. Agreement and Associations between Countermovement Jump, 5-Time Sit-To-Stand, Lower-Limb Muscle Power Equations, and Physical Performance Tests in Community-Dwelling Older Adults. J Clin Med. 2024;13(12):3380. doi:10.3390/jcm13123380

S14. Guralnik JM, Simonsick EM, Ferrucci L, et al. A short physical performance battery assessing lower extremity function: association with self-reported disability and prediction of mortality and nursing home admission. J Gerontol. 1994;49(2):M85-94. doi:10.1093/geronj/49.2.m85

S15. McCarthy EK, Horvat MA, Holtsberg PA, Wisenbaker JM. Repeated chair stands as a measure of lower limb strength in sexagenarian women. J Gerontol A Biol Sci Med Sci. 2004;59(11):1207-1212. doi:10.1093/gerona/59.11.1207

S16. Drouin JM, Valovich-mcLeod TC, Shultz SJ, Gansneder BM, Perrin DH. Reliability and validity of the Biodex system 3 pro isokinetic dynamometer velocity, torque and position measurements. Eur J Appl Physiol. 2004;91(1):22-29. doi:10.1007/s00421-003-0933-0

S17. Snyder-Mackler L. Isokinetics in Human Performance: In: Medicine and Science in Sports and Exercise. Vol 32. ; 2000:2153. doi:10.1097/00005768-200012000-00034

S18. Dafoe W. Principles of Exercise Testing and Interpretation. Can J Cardiol. 2007;23(4):274.

S19. van der Steeg GE, Takken T. Reference values for maximum oxygen uptake relative to body mass in Dutch/Flemish subjects aged 6-65 years: the LowLands Fitness Registry. Eur J Appl Physiol. 2021;121(4):1189-1196. doi:10.1007/s00421-021-04596-6

**Figures**

**Figure S1. Flow diagram depicting data availability.**

**
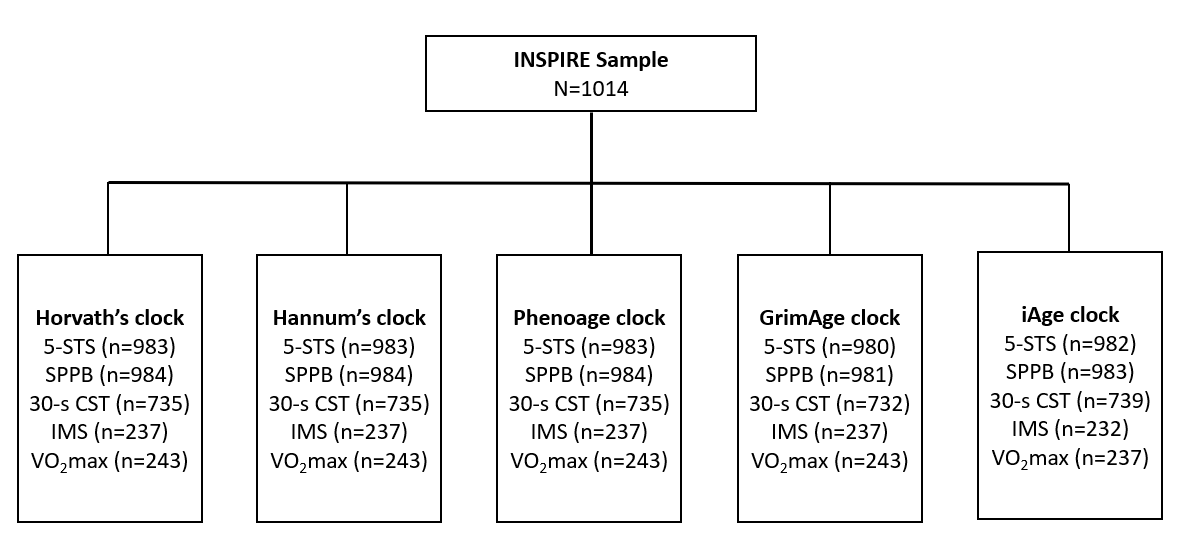
**

**Tables**

**Table S1.** Comparison of the characteristics of included individuals based on the availability of data on VO2max/IMS

| **Characteristics** | **Whole sample**  N=1014 | **Sample without data on**  **V̇O2max/IMS**  N=769 | **Sample with data on V̇O2max/IMS**  N=245 | **p-value** |
| --- | --- | --- | --- | --- |
| **Women, No. (%)** | 637 (62.82%) | 498 (64.76%) | 139 (56.73%) | 0.024 |
| **Age,** y | 64 (49-78) | 64 (48-79) | 65 (52-72) | 0.405 |
| **Height,** m | 1.65 (0.10) | 1.65 (0.10) | 1.66 (0.10) | 0.252 |
| **Weight,** kg | 68.83 (14.12) | 68.44 (14.03) | 70.02 (14.38) | 0.128 |
| **Body Mass Index,** kg . m^-2^ | 25.06 (4.29) | 24.99 (4.38) | 25.26 (3.98) | 0.393 |
| **Charlson Index** | 2 (1-4) | 2 (0-4) | 2 (1-3) | 0.205 |
| **Smoking Index** | 0 (0-35) | 0 (0-27) | 0 (0-71) | 0.273 |
| **Horvath’s DNAm Age,** y | 60.09 (15.42) | 60.03 (16.21) | 60.31 (12.70) | 0.805 |
| **Hannum’s DNAm Age,** y | 50.81 (15.30) | 50.72 (16.06) | 51.03 (13.65) | 0.787 |
| **PhenoAge DNAm Age,** y | 46.25 (17.87) | 46.26 (18.75) | 46.20 (14.79) | 0.961 |
| **GrimAge DNAm Age,** y | 61.17 (15.23) | 60.97 (15.89) | 61.78 (12.97) | 0.470 |
| **iAGe Age, y** | 58.01 (9.18) | 58.31 (9.22)) | 57.05 (8.99) | 0.064 |
| **5-STS, s** | 9.05 (2.88) | 9.03 (2.99) | 9.10 (2.49) | 0.721 |
| **SPPB scorea** | 12 (12-12) | 12 (12-12) | 12 (12-12) | 0.501 |
| **30-s CST,** n | 15.83 (4.70) | 15.73 (4.75) | 16.78 (4.18) | 0.072 |

**Abbreviations:** DNAm: DNA methylation, ISM: isokinetic muscle strength, SPPB: Short Physical Performance Battery, V̇O2max: maximum oxygen uptake, 5-STS: five-times sit-to-stand test, 30-s CST: 30-second chair stand test.

**Table S2.** Independent and interactive association of BAA and age (or age^2^) and physical capacities in the INSPIRE sample.

Bold P values indicate statistical significance.

IV:independent variable.

| **Horvath's BAA** | | | | | | | | |
| --- | --- | --- | --- | --- | --- | --- | --- | --- |
| **5-STS (n=983)** | | | | | **SPPB** | | | |
| IV | β | 95%CI LL | 95% CI UL | p-value | β | 95%CI LL | 95% CI UL | p-value |
| Age | -0.0432781 | -0.0968348 | 0.0102785 | 0.113 | 0.1083778 | 0.0832164 | 0.1335393 | P<0.001 |
| Age^2^ | 0.0007311 | 0.0002342 | 0.0012279 | 0.004 | -0.0011385 | -0.0013705 | -0.0009065 | P<0.001 |
| Horvath’s | 0.3102413 | -0.9016456 | 1.522128 | 0.615 | 0.3933118 | -0.174311 | 0.9609345 | 0.174 |
| Age x Horvath’s | -0.0109706 | -0.0590266 | 0.0370854 | 0.654 | -0.020253 | -0.0425767 | 0.0020707 | 0.075 |
| Age^2^ x Horvath’s | 0.0000936 | -0.0003363 | 0.0005235 | 0.669 | 0.0001935 | -0.00000497 | 0.000392 | **0.056** |
| **30-s CST** | | | | |  |  |  |  |
| IV | β | 95%CI LL | 95% CI UL | p-value |  |  |  |  |
| Age | 0.0367391 | -0.0548007 | 0.128279 | 0.431 |  |  |  |  |
| Age^2^ | -0.0010267 | -0.0018905 | -0.0001629 | 0.02 |  |  |  |  |
| Horvath’s | -0.4361115 | -2.501354 | 1.629131 | 0.679 |  |  |  |  |
| Age x Horvath’s | 0.0055866 | -0.0793878 | 0.0905611 | 0.897 |  |  |  |  |
| Age^2^ x Horvath’s | -0.0000568 | -0.0008287 | 0.000715 | 0.885 |  |  |  |  |
| **VO2 MAX** | | | | | **IMS** | | | |
| IV | β | 95%CI LL | 95% CI UL | p-value | β | 95%CI LL | 95% CI UL | p-value |
| Age | -0.2496336 | -0.3353251 | -0.163942 | P<0.001 | -0.7943028 | -1.319147 | -0.2694585 | 0.003 |

| Horvath’s | -2.120715 | -5.64321 | 1.401779 | 0.236 | -5.687971 | -26.96504 | 15.5891 | 0.599 |
| --- | --- | --- | --- | --- | --- | --- | --- | --- |
| Age x Horvath’s | 0.0263127 | -0.0285109 | 0.0811363 | 0.345 | 0.0616789 | -0.2711249 | 0.3944827 | 0.715 |

| **Hannum’s BAA** | | | | | | | | |
| --- | --- | --- | --- | --- | --- | --- | --- | --- |
| **5-STS (n=983)** | | | | | **SPPB** | | | |
| IV | β | 95%CI LL | 95% CI UL | p-value | β | 95%CI LL | 95% CI UL | p-value |
| Age | -0.0419 | -0.0947 | 0.01086 | 0.119 | 0.1064 | 0.08164 | 0.13117 | P<0.001 |
| Age^2^ | 0.00072 | 0.00022 | 0.00121 | 0.004 | -0.0011 | -0.0013 | -0.0009 | P<0.001 |
| **Hannum’s** | 0.15059 | -1.0249 | 1.32607 | 0.802 | 0.52286 | -0.027 | 1.07267 | 0.062 |
| Age x  **Hannum’s** | 0.00287 | -0.0434 | 0.04915 | 0.903 | -0.0251 | -0.0466 | -0.0037 | 0.022 |
| Age^2^ x  **Hannum’s** | -8E-05 | -0.0005 | 0.00034 | 0.708 | 0.00025 | 5.8E-05 | 0.00044 | **0.011** |
| **30-s CST** | | | | |  |  |  |  |
| IV | β | 95%CI LL | 95% CI UL | p-value |  |  |  |  |
| Age | 0.0248 | -0.0655 | 0.11508 | 0.59 |  |  |  |  |
| Age^2^ | -0.0009 | -0.0018 | -5E-05 | 0.037 |  |  |  |  |
| **Hannum’s** | -1.0346 | -2.9784 | 0.9093 | 0.296 |  |  |  |  |
| Age x  **Hannum’s** | 0.02666 | -0.0515 | 0.10485 | 0.503 |  |  |  |  |
| Age^2^ x  **Hannum’s** | -0.0002 | -0.0009 | 0.00052 | 0.611 |  |  |  |  |
| **VO2 MAX** | | | | | **IMS** | | | |
| IV | β | 95%CI LL | 95% CI UL | p-value | β | 95%CI LL | 95% CI UL | p-value |
| Age | -0.2617 | -0.3477 | -0.1756 | P<0.001 | -0.7522 | -1.278 | -0.2263 | 0.005 |
| **Hannum’s** | -0.9844 | -4.8768 | 2.90813 | 0.618 | -15.628 | -39.109 | 7.85251 | 0.191 |
| Age x  **Hannum’s** | 0.02405 | -0.0332 | 0.08128 | 0.408 | 0.19422 | -0.1526 | 0.54107 | 0.271 |

| **PhenoAge BAA** | | | | | | | | |
| --- | --- | --- | --- | --- | --- | --- | --- | --- |
| **5-STS (n=983)** | | | | | **SPPB** | | | |
| IV | β | 95%CI LL | 95% CI UL | p-value | β | 95%CI LL | 95% CI UL | p-value |
| Age | -0.0443 | -0.0975 | 0.00895 | 0.103 | 0.1064 | 0.08164 | 0.13117 | P<0.001 |
| Age^2^ | 0.00074 | 0.00025 | 0.00124 | 0.003 | -0.0011 | -0.0013 | -0.0009 | P<0.001 |
| **PhenoAge** | 0.53556 | -0.7059 | 1.77706 | 0.397 | 0.52286 | -0.027 | 1.07267 | 0.062 |
| Age x  **PhenoAge** | -0.0198 | -0.0693 | 0.02966 | 0.432 | -0.0251 | -0.0466 | -0.0037 | 0.022 |
| Age^2^ x  **PhenoAge** | 0.00017 | -0.0003 | 0.00062 | 0.458 | 0.00025 | 5.8E-05 | 0.00044 | **0.011** |
| **30-s CST** | | | | |  |  |  |  |
| IV | β | 95%CI LL | 95% CI UL | p-value |  |  |  |  |
| Age | 0.03283 | -0.0577 | 0.12338 | 0.477 |  |  |  |  |
| Age^2^ | -0.001 | -0.0018 | -0.0001 | 0.026 |  |  |  |  |
| **PhenoAge** | -1.1918 | -3.2484 | 0.86483 | 0.256 |  |  |  |  |
| Age x  **PhenoAge** | 0.02794 | -0.0555 | 0.11134 | 0.511 |  |  |  |  |
| Age^2^ x  **PhenoAge** | -0.0002 | -0.0009 | 0.00057 | 0.628 |  |  |  |  |
| **VO2 MAX** | | | | | **IMS** | | | |
| IV | β | 95%CI LL | 95% CI UL | p-value | β | 95%CI LL | 95% CI UL | p-value |
| Age | -0.2502 | -0.3362 | -0.1642 | P<0.001 | -0.7994 | -1.3261 | -0.2727 | 0.003 |
| **PhenoAge** | -1.2074 | -4.6214 | 2.20662 | 0.486 | -0.7199 | -21.538 | 20.0979 | 0.946 |
| Age x  **PhenoAge** | 0.01008 | -0.0428 | **0.063** | 0.707 | -0.0354 | -0.3576 | 0.28688 | 0.829 |

| **GrimAge BAA** | | | | | | | | |
| --- | --- | --- | --- | --- | --- | --- | --- | --- |
| **5-STS (n=983)** | | | | | **SPPB** | | | |
| IV | β | 95%CI LL | 95% CI UL | p-value | β | 95%CI LL | 95% CI UL | p-value |
| Age | -0.0499 | -0.103 | 0.00324 | 0.066 | 0.1099 | 0.08483 | 0.13497 | P<0.001 |
| Age^2^ | 0.00083 | 0.00033 | 0.00132 | 0.001 | -0.0012 | -0.0014 | -0.0009 | P<0.001 |
| **GrimAge** | 2.11298 | 0.24728 | 3.97868 | 0.026 | -0.1566 | -1.0231 | 0.70994 | 0.723 |
| Age x  **GrimAge** | -0.075 | -0.141 | -0.0091 | 0.026 | 0.00749 | -0.0228 | 0.03781 | 0.628 |
| Age^2^ x  **GrimAge** | 0.00066 | 0.00011 | 0.00122 | **0.019** | -0.0001 | -0.0004 | 0.00015 | 0.429 |
| **30-s CST** | | | | |  |  |  |  |
| IV | β | 95%CI LL | 95% CI UL | p-value |  |  |  |  |
| Age | 0.04034 | -0.0515 | 0.1322 | 0.389 |  |  |  |  |
| Age^2^ | -0.0011 | -0.0019 | -0.0002 | 0.016 |  |  |  |  |
| **GrimAge** | -4.0001 | -7.2363 | -0.7639 | 0.015 |  |  |  |  |
| Age x  **GrimAge** | 0.12484 | 0.00916 | 0.24051 | 0.034 |  |  |  |  |
| Age^2^ x  **GrimAge** | -0.001 | -0.0019 | 5.8E-06 | **0.051** |  |  |  |  |
| **VO2 MAX** | | | | | **IMS** | | | |
| IV | β | 95%CI LL | 95% CI UL | p-value | β | 95%CI LL | 95% CI UL | p-value |
| Age | -0.2544 | -0.3379 | -0.1709 | P<0.001 | -0.8117 | -1.333 | -0.2904 | 0.002 |
| **GrimAge** | -0.5728 | -3.2013 | 2.05579 | 0.668 | 8.78048 | -7.7777 | 25.3387 | 0.297 |
| Age x  **GrimAge** | -0.009 | -0.0521 | 0.03409 | 0.68 | -0.1278 | -0.4016 | 0.14591 | 0.358 |

| **iAge BAA** | | | | | | | | |
| --- | --- | --- | --- | --- | --- | --- | --- | --- |
| **5-STS (n=983)** | | | | | **SPPB** | | | |
| IV | β | 95%CI LL | 95% CI UL | p-value | β | 95%CI LL | 95% CI UL | p-value |
| Age | -0.0272 | -0.083 | 0.02871 | 0.34 | 0.1115 | 0.083 | 0.139 | P<0.001 |
| Age^2^ | 0.00058 | 6.9E-05 | 0.0011 | 0.026 | -0.001 | -0.0012 | -0.0008 | P<0.001 |
| **iAge** | 1.02467 | -0.5502 | 2.59958 | 0.202 | -1.791 | -2.516 | -1.066 | P<0.001 |
| Age x **iAge** | -0.0499 | -0.1084 | 0.00859 | 0.094 | 0.072 | 0.046 | 0.097 | P<0.001 |
| Age^2^ x **iAge** | 0.00051 | 8.5E-06 | 0.00102 | 0.046 | -0.0007 | -0.0008 | -0.0004 | P<0.001 |
| **30-s CST** | | | | |  |  |  |  |
| IV | β | 95%CI LL | 95% CI UL | p-value |  |  |  |  |
| Age | 0.0193 | -0.0746 | 0.11319 | 0.687 |  |  |  |  |
| Age^2^ | -0.0008 | -0.0017 | 6.1E-05 | 0.068 |  |  |  |  |
| **iAge** | 0.29265 | -2.3485 | 2.93382 | 0.828 |  |  |  |  |
| Age x **iAge** | 0.01478 | -0.0846 | 0.11417 | 0.77 |  |  |  |  |
| Age^2^ x **iAge** | -0.0003 | -0.0012 | 0.00054 | 0.463 |  |  |  |  |
| **VO2 MAX** | | | | | **IMS** | | | |
| IV | β | 95%CI LL | 95% CI UL | p-value | β | 95%CI LL | 95% CI UL | p-value |
| Age | -0.2429 | -0.3396 | -0.1462 | P<0.001 | -0.845 | -1.4237 | -0.2664 | 0.004 |
| **iAge** | 1.58382 | -0.6526 | 3.82023 | 0.164 | 5.53457 | -7.8048 | 18.8739 | 0.414 |
| Age x **iAge** | -0.0222 | -0.0588 | 0.01435 | 0.232 | -0.1338 | -0.3526 | 0.08496 | 0.229 |

**Table S3. SPPB scores by age group.**

| **Age range** | **N** | **Mean SPPB score** | **Range** |
| --- | --- | --- | --- |
| 20-29 years | 80 | 11.97 | 11-12 |
| 30-39 years | 87 | 11.97 | 10-12 |
| 40-49 years | 103 | 11.94 | 10-12 |
| 50-59 years | 140 | 11.88 | 8-12 |
| 60-69 years | 184 | 11.77 | 7-12 |
| 70-79 years | 202 | 11.40 | 2-12 |
| 80-89 years | 183 | 10.66 | 2-12 |
| ≥90 years | 17 | 7.3 | 1-12 |

**Table S4. Mean absolute error, median absolute error and root mean square error of the biological age estimated by biological clocks and chronological age in our sample.**

| **Biological Clock** | **Mean absolute error ± SD** | **Median absolute error (IQR)** | **Root mean square of error** |
| --- | --- | --- | --- |
| **Horvath’s DNAm Age,** y | -1.37 ± 6.48 | -1.03 (-5.54, 3.11) | 6.62 |
| **Hannum’s DNAm Age,** y | -10.65 ± 5.83 | -10.49 (-14.89, -6.63) | 12.14 |
| **PhenoAge DNAm Age,** y | -15.21 ± 5.93 | -15.14 (-19.19, -11.39) | 16.33 |
| **GrimAge DNAm Age,** y | -0.32 ± 5.21 | -0.76 (-4.16, 3.34) | 5.22 |
| **iAge, y** | -3.34 ± 16.8 | -5.64 (-16.02, 7.92) | 17.08 |

**SD: Standard deviation; IQR: interquartile range.**

**Table S5. Associations between biological age acceleration according to different biological clocks and SPPB in participants ≥60 years**

|  |  | **SPPB** | |
| --- | --- | --- | --- |
| **BAA** | n | β (95% CI) | p-value |
| **Horvath’s** |  |  |  |
| Model 1 | 577 | -0.02 (-0.14, 0.10) | 0.771 |
| Model 2 | 576 | -0.03 (-0.14, 0.10) | 0.648 |
| **Hannum’s** |  |  |  |
| Model 1 | 577 | 0.02 (-0.10, 0.15) | 0.720 |
| Model 2 | 576 | 0.01 (-0.11, 0.13) | 0.897 |
| **PhenoAge** |  |  |  |
| Model 1 | 577 | -0.03 (-0.15, 0.09) | 0.650 |
| Model 2 | 576 | -0.01 (-0.13, 0.11) | 0.850 |
| **GrimAge** |  |  |  |
| Model 1 | 577 | **-0.15 (-0.27, 0.03)** | **0.018** |
| Model 2 | 576 | -0.13 (-0.26, 0.00) | 0.055 |
| **iAge** |  |  |  |
| Model 1 | 576 | -0.04 (-0.17, 0.09) | 0.524 |
| Model 2 | 575 | -0.09 (-0.21, 0.03) | 0.156 |

**Abbreviations:** DNAm: DNA methylation, SPPB: Short Physical Performance Battery, 5-STS: five-times sit-to-stand test, 30-s CST: 30-second chair stand test.

1. β-coefficient correspond to the increase in 1-SD in the BAA
2. Significant associations are displayed in bold.
